# Thoracic Actinomycosis Mimicking Tuberculosis Presenting as Pneumonia and a Chest Wall Mass

**DOI:** 10.7759/cureus.117069

**Published:** 2026-09-28

**Authors:** Mohsin Fayyaz, M A Sayed Joy, Manasi Limaye, Shaheryar Khan, Muhammad Ali

**Affiliations:** 1 Medicine, Salford Royal NHS Foundation Trust, Manchester, GBR; 2 Neurology, Salford Royal Hospital, Manchester, GBR; 3 Diabetes and Endocrinology, The Christie NHS Foundation Trust, Manchester, GBR; 4 Emergency Medicine, Darlington Memorial Hospital, Darlington, GBR; 5 Internal Medicine, Salford Royal NHS Foundation Trust, Manchester, GBR

**Keywords:** actinomyces, actinomycosis, aggregatibacter actinomycetemcomitans, atypical pneumonia, chest wall abscess, chest wall mass, eikenella corrodens, pulmonary actinomycosis, pulmonary infection, tuberculosis

## Abstract

Thoracic actinomycosis is an uncommon chronic infection caused by *Actinomyces* species. It can produce pulmonary consolidation and extend across tissue planes into the chest wall, closely mimicking tuberculosis or malignancy.

A previously well woman in her 20s presented with 2-3 weeks of left posterolateral chest wall pain, progressive swelling, weight loss of 2-3 kg, cough and haemoptysis. She had had two earlier episodes of left-sided pneumonia treated with short antibiotic courses. Examination showed a large (>10 cm), warm, firm, non-fluctuant swelling over the left posterolateral chest wall. Blood tests showed a marked inflammatory response (white blood cell count (WBC): 16.2 × 10⁹/L, C-reactive protein (CRP): 168-169 mg/L) and microcytic anaemia. CT demonstrated superoposterior left lower lobe consolidation with perifissural nodules, volume loss, reactive lymph nodes and a contiguous chest wall collection. Tuberculosis was considered first. Ultrasound-guided biopsy showed a heterogeneous, septated mass with necrosis. Acid-fast bacillus (AFB) staining, tuberculosis polymerase chain reaction (PCR) and mycobacterial culture at eight weeks were negative. Histology showed granulation tissue and abscess with filamentous Gram-positive aggregates suggestive of *Actinomyces*. *Actinomyces* was not isolated in culture. She improved on intravenous co-amoxiclav and completed a prolonged oral β-lactam course, later changed to cefalexin after microbiology review.

This case illustrates the difficulty of diagnosing thoracic actinomycosis when it presents with contiguous pulmonary and chest wall disease. Histology supported a presumptive diagnosis. Actinomycosis should be considered when chronic pulmonary infection extends into the chest wall, particularly after tuberculosis has become substantially less likely.

## Introduction

Thoracic actinomycosis is an uncommon infection caused by *Actinomyces*, anaerobic or microaerophilic Gram-positive filamentous commensals of the oropharynx and gastrointestinal tract [1,2]. Disease may follow disruption of mucosal barriers. Predisposing factors include poor oral hygiene, dental disease, aspiration, smoking and underlying lung disease, although immunocompetent patients without these risks can also be affected [2,3].

Pulmonary infection can spread across anatomical planes into the pleura and chest wall, producing a mass or collection that closely resembles tuberculosis or malignancy [1,3,4]. Culture is often negative, particularly after prior antibiotics, so histology showing filamentous Gram-positive aggregates may be needed to support the diagnosis [2,4].

We report a 23-year-old woman with a large left chest wall collection, left lower lobe consolidation and constitutional symptoms in whom tuberculosis was the leading initial concern. The case highlights the difficulty of distinguishing thoracic actinomycosis from tuberculosis when *Actinomyces* is not isolated in culture.

## Case presentation

A 23-year-old woman, previously fit and well, presented with a 2-3-week history of left posterolateral chest wall pain followed by gradually increasing swelling. She described feeling cold intermittently, fever and night sweats. She reported an unintentional weight loss of approximately 2-3 kg. During the few days preceding admission, she also reported cough and haemoptysis. Approximately one month before presentation, she had fallen from a scooter and struck her back, although she did not sustain a significant injury and reported no swelling or substantial bruising immediately following the incident. She had no known history of tuberculosis and reported no known close household contact with active tuberculosis. There was no significant past medical history, and she was not taking regular medications. She had previously smoked but had stopped approximately one year before presentation. Of particular relevance, she had experienced two previous episodes of illness earlier in the year that had been treated as pneumonia. Both episodes were associated with left-sided/posterior chest pain, cough and haemoptysis and were treated with approximately one week of antibiotics.

On examination, she was alert and orientated. She was initially haemodynamically stable, although her blood pressure was recorded as 99/62 mmHg (Table 1). Respiratory examination demonstrated reduced air entry at the left base without crepitations or wheeze. There was a large, approximately 10 cm or greater, relatively hard and non-fluctuant swelling over the left lateral/posterior chest wall. The swelling was warm and tender. There was no palpable cervical or axillary lymphadenopathy. Cardiovascular examination was otherwise unremarkable apart from intermittent tachycardia (Table 1).

**Table 1:** NEWS observations The highest NEWS score in the last 24 hours was 2 (early morning), mainly due to low blood pressure (99/62 mmHg). After two hours, the score improved to 1, with better blood pressure (121/79 mmHg) but a higher heart rate (110 bpm); the patient remained alert and stable on air throughout. BP: blood pressure, NEWS: National Early Warning Score Source: [4]

Parameter	On presentation	After 2 hours
NEWS score	2	1
Respiration rate (resp/min)	14	18
O2 device or air	A - air	A - air
O2 saturation (%)	100	100
Oxygen flow rate (L/min)	0	0
Temperature (°C)	36.8	37.4
BP systolic (mmHg)	99	121
BP diastolic (mmHg)	62	79
Heart rate (bpm)	78	110
Level of consciousness	A - alert	A - alert

Physiological observations were recorded using the National Early Warning Score (NEWS/NEWS2), developed by the Royal College of Physicians as a standardised track-and-trigger system for acute illness. The highest NEWS score in the preceding 24 hours was 2, mainly because of hypotension (99/62 mmHg). After two hours, the score improved to 1, with better blood pressure (121/79 mmHg) but a higher heart rate (110 bpm). The patient remained alert and stable on air throughout.

Initial laboratory investigations demonstrated a significant inflammatory response, with a white blood cell count (WBC) of 16.2 × 10⁹/L, neutrophils of 12.9 × 10⁹/L, haemoglobin of 96 g/L and mean corpuscular volume of 77 fL (Table 2).

**Table 2:** Haematology (full blood count) On admission, there was marked neutrophilia (WBC: 16.2-19.0 × 10⁹/L, neutrophils: 12.9-15.5 × 10⁹/L) and reactive thrombocytosis (platelets: 639-710 × 10⁹/L), consistent with a significant systemic inflammatory response associated with an active infective process. Serial blood counts showed progressive recovery of haemoglobin to 126 g/L and rapid normalisation of white cells (7.8-8.2 × 10⁹/L), neutrophils (3.8-5.8 × 10⁹/L) and platelets (389-420 × 10⁹/L) after antibiotics were started, providing objective evidence of a sustained response to antimicrobial therapy. * indicates a result outside the local adult laboratory reference range. MCV: mean corpuscular volume, WBC: white blood cell count

Parameter	Admission	Day 2	Discharge	4-month follow-up	Reference range
Haemoglobin (g/L)	96*	105	95*	126	115-165
MCV (fL)	77.0*	78.0	77.0*	80.0	80-100
WBC (×10⁹/L)	16.2*	19.0*	8.7	8.2	4.0-11.0
Neutrophils (×10⁹/L)	12.9*	15.5*	5.8	3.8	2.0-7.5
Lymphocytes (×10⁹/L)	2.1	2.3	2.1	3.6	1.0-4.0
Platelets (×10⁹/L)	639*	710*	699*	389	150-400

On admission, C-reactive protein (CRP) was 168 mg/L. Renal function tests (RFTs), electrolytes (E) and liver function tests (LFTs) were normal (Table 3). 

**Table 3:** Biochemistry results CRP was markedly elevated on admission (168 → peak 186 mg/L), consistent with significant bacterial infection or abscess, and fell steeply to 72 mg/L within days, then to 2.0 mg/L on four-month follow-up. Mild protein abnormalities (raised globulins, borderline low albumin) improved over time, while renal function, electrolytes and liver enzymes remained largely normal. The dramatic and sustained fall in CRP, together with subsequent clinical resolution, supported successful control of the underlying infective process following prolonged antimicrobial therapy. * indicates a result outside the local adult laboratory reference range. ALT: alanine aminotransferase, CRP: C-reactive protein

Parameter	Admission	Day 2	Discharge	4-month follow-up	Reference range
Sodium (mmol/L)	137	139	138	136	133-146
Potassium (mmol/L)	4.6	4.6	4.4	4.1	3.5-5.3
Urea (mmol/L)	4.7	3.7	2.3*	5.3	2.5-7.8
Creatinine (µmol/L)	58	46	44	62	45-84 (female)
Albumin (g/L)	40	39	39	48	35-50
ALT (U/L)	17	10	13	18	<40
CRP (mg/L)	168*	186*	72*	2.0	<5

Coronal view (Figure 1) and transverse view (Figure 2) of CT of the abdomen and pelvis with contrast demonstrated a soft tissue lesion in the left posterior thoracolumbar region with associated underlying left lower lobe consolidation.

**Figure 1:**
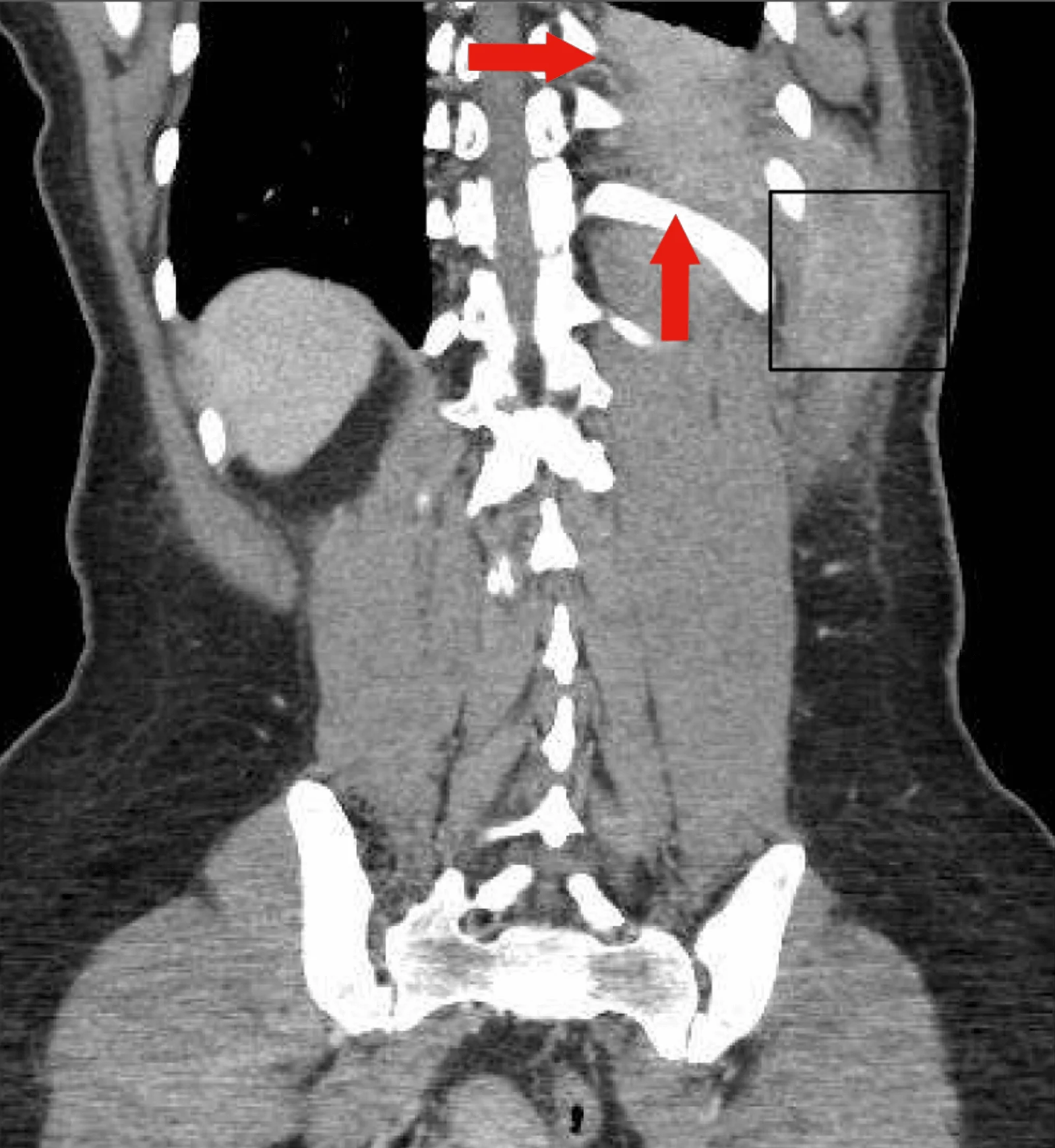
CT of the abdomen and pelvis (coronal view) CT showed a heterogeneous, peripherally enhancing soft tissue collection with central lucency in the left posterior thoracolumbar chest wall, abutting the paraspinal muscles, together with underlying left lower lobe collapse and consolidation. No bone destruction or intra-abdominal abnormality was seen. Red arrows indicate the consolidation; the square box indicates the chest wall collection.

**Figure 2:**
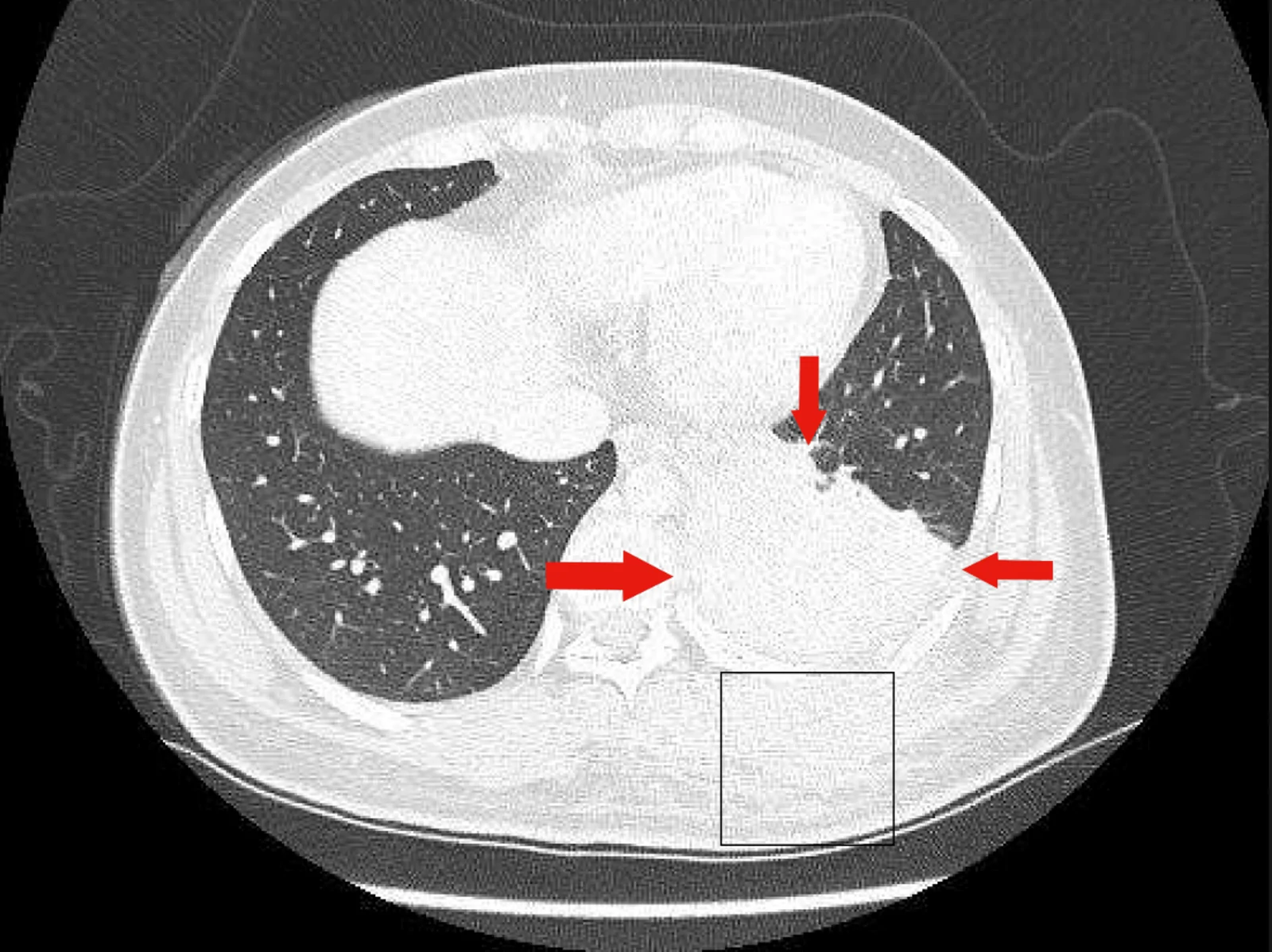
CT of the abdomen and pelvis (transverse view) CT showed a heterogeneous, peripherally enhancing soft tissue collection with central lucency in the left posterior thoracolumbar chest wall, abutting the paraspinal muscles, together with underlying left lower lobe collapse and consolidation. No bone destruction or intra-abdominal abnormality was seen. Red arrows indicate the consolidation; the square box indicates the chest wall collection.

Subsequent CT of the chest demonstrated dense consolidation in the superoposterior left lower lobe with adjacent scattered perifissural nodules, considered likely inflammatory, together with associated left lower lobe volume loss (Figure 3). A partially visualised peripherally enhancing left flank/lower thoracic chest wall lesion/collection was also present.

**Figure 3:**
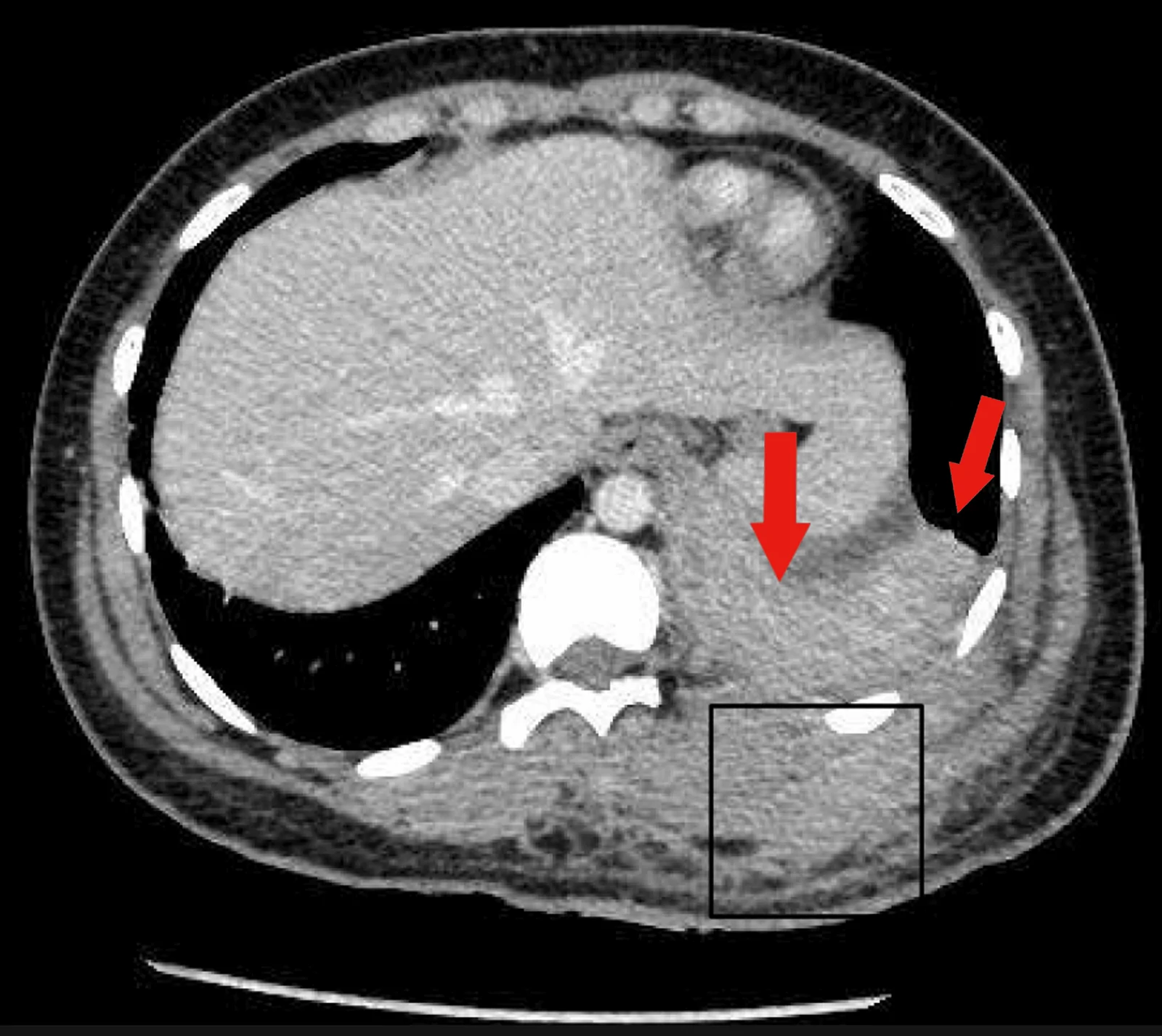
CT of the thorax with contrast Chest CT showed consolidation in the superoposterior left lower lobe with adjacent perifissural nodules and volume loss in the left lower lobe, together with reactive lymph nodes. No specific radiological features of pulmonary tuberculosis were identified; further microbiological investigation for atypical infection was advised. The previously noted peripherally enhancing collection in the left flank and lower thoracic chest wall remained visible, with no destructive bone lesions. Red arrows indicate the consolidation; the white box indicates the chest wall collection.

Given the history of travel, weight loss, haemoptysis, pulmonary abnormalities and chest wall collection, tuberculosis was considered an important diagnosis to exclude. An ultrasound-guided biopsy was performed, which demonstrated a heterogeneous, septated mass containing areas of necrosis and collections.

Sputum acid-fast bacillus (AFB) samples were negative. AFB microscopy of the chest wall material demonstrated no acid-fast bacilli. *Mycobacterium tuberculosis* complex DNA was not detected by PCR performed at the regional reference laboratory. Liquid mycobacterial culture was negative at six weeks and remained negative for mycobacteria at eight weeks. HIV and hepatitis screening were negative. Urinary *Legionella* and pneumococcal antigens were also negative.

The chest wall necrotic material demonstrated numerous white blood cells on Gram film, with no organisms seen on direct microscopy. Two organisms were subsequently reported in enrichment culture: *Aggregatibacter actinomycetemcomitans* and *Eikenella corrodens* (both enrichment only) (Table 4). The clinical significance of these organisms was considered in conjunction with the histological findings and clinical presentation. The microbiological findings were not considered diagnostic of tuberculosis.

**Table 4:** Microbiology cultures of the aspirate Chest wall aspirate Gram films showed numerous white cells but no organisms. Culture (enrichment only) grew *Aggregatibacter actinomycetemcomitans* from the first sample and *Eikenella corrodens* from the second; *Actinomyces* itself was not isolated. Although *Actinomyces* itself was not isolated, *Aggregatibacter actinomycetemcomitans* and *Eikenella corrodens* were recovered from enrichment cultures. Both are oral commensals that may occur in polymicrobial infections involving *Actinomyces*; however, their isolation alone is not diagnostic of actinomycosis. In this case, their recovery from necrotic material, together with the characteristic histological findings and contiguous pulmonary and chest wall involvement, provided supportive evidence for an oral-source polymicrobial infection with features strongly suggestive of thoracic actinomycosis.

Parameter	1st culture: necrotic mass aspirate	2nd culture: necrotic aspirate
Gram film	White blood cells +++, no organisms seen	White blood cells +++, no organisms seen
Organism isolated	*Aggregatibacter actinomycetemcomitans* (enrichment only)	*Eikenella corrodens* (enrichment only)
Quantity	Enrichment only	Enrichment only

Histological examination demonstrated fragments of granulation tissue and abscess formation with possible *Actinomyces*-like organisms (Table 5 and Figure 4). There were no granulomas and no evidence of malignancy. Special stains were subsequently performed. The areas containing filamentous aggregates demonstrated positive staining with Gram stain, suggestive of *Actinomyces*. Periodic acid-Schiff with diastase (PAS-D) and Ziehl-Neelsen (ZN) staining did not demonstrate definite microorganisms. The final histopathology report concluded that the filamentous aggregates were suggestive of *Actinomyces *(Figure 4).

**Table 5:** Histology and microbiology results (chest wall mass) Histology of the chest wall cores showed granulation tissue and an abscess with possible *Actinomyces*-like organisms; no granulomas or malignancy were seen. Special stains confirmed filamentous aggregates that were Gram-positive, suggestive of *Actinomyces*, while PAS-D and ZN were negative. The findings were strongly suggestive of actinomycosis and were interpreted in conjunction with the clinical, radiological and microbiological findings. PAS-D: Periodic acid-Schiff with diastase, ZN: Ziehl-Neelsen

Section	Findings
Specimen	Chest wall mass: several needle cores measuring 1-5 mm in maximum diameter; all used
Macroscopy	Several needle cores measuring 1-5 mm in maximum diameter; all used
Microscopy	Fragments of granulation tissue/abscess with possible *Actinomyces*-like organisms; there are no granulomas and no evident malignancy
Special stains requested	PAS-D, Gram stain, ZN; supplementary report follows
Supplementary report	The area with filamentous aggregates shows positive staining on Gram stain, suggestive of *Actinomyces*; PAS-D and ZN reveal no definite microorganisms; please correlate with clinical and microbiological parameters

**Figure 4:**
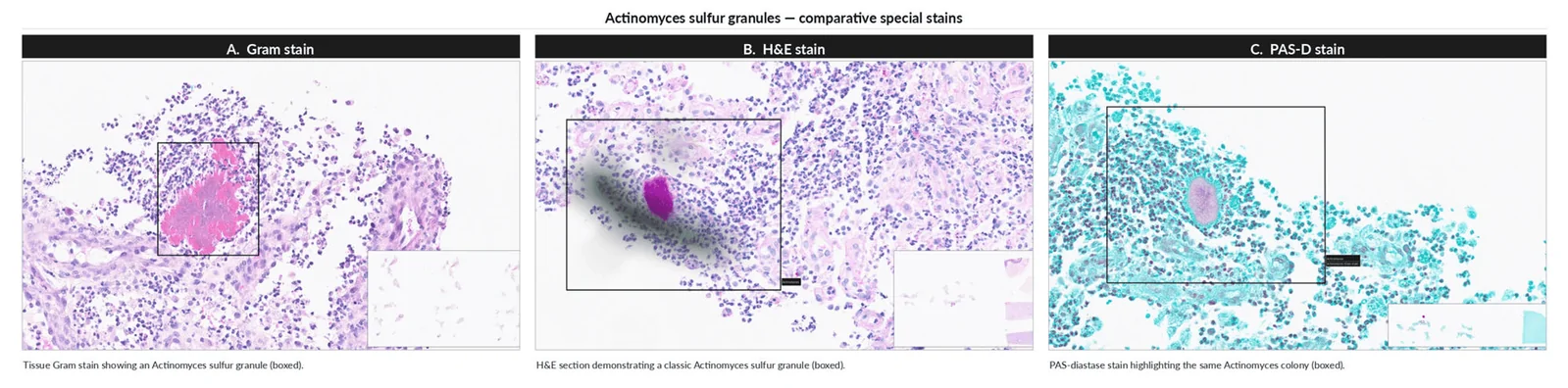
Histology A: Gram stain: tissue Gram stain showing an *Actinomyces* sulfur granule (boxed). B: H&E stain: H&E section demonstrating a classic *Actinomyces* sulfur granule (boxed). C: PAS-D stain: PAS-D stain highlighting the same *Actinomyces* colony (boxed). H&E: haematoxylin and eosin, PAS-D: periodic acid-Schiff with diastase

The patient was initially treated with intravenous co-amoxiclav 1.2 g for two days, with subsequent clinical improvement. She was then discharged on oral co-amoxiclav 625 mg three times daily, which was continued for eight weeks. Following discussion with the microbiology team, treatment was subsequently changed to oral cefalexin 1 g twice daily because it would cover both *Actinomyces* and the cultured *Eikenella*, and this was continued for a further four months because of the extensive pulmonary and chest wall involvement. The patient remained under clinical and microbiological follow-up.

The patient’s inflammatory markers improved substantially following the initiation of antimicrobial therapy, and the chest wall mass resolved on follow-up visit. The prolonged antimicrobial course was determined in conjunction with the microbiology team because of the extensive chest wall involvement and associated pulmonary disease.

## Discussion

This case illustrates the diagnostic difficulty of thoracic actinomycosis presenting as a large chest wall collection with underlying pulmonary consolidation. The infection classically follows aspiration of oropharyngeal organisms [1,3]. It is characterised by spread across anatomical planes into the pleura, the chest wall or, less often, the spine [2,3]. The contiguous left lower lobe consolidation and posterior chest wall collection in this patient were typical of that behaviour and raised the possibility of actinomycosis once tuberculosis had become substantially less likely.

The presentation closely mimicked tuberculosis. Recurrent left-sided pneumonia, haemoptysis, weight loss and a chest wall mass appropriately prompted extensive mycobacterial investigation [1,3]. Negative sputum and tissue acid-fast bacillus microscopy, negative tuberculosis PCR and negative mycobacterial culture at eight weeks, together with the absence of granulomas on histology, made tuberculosis much less likely and allowed other diagnoses to be considered. These results do not completely exclude tuberculosis.

Histology supported the diagnosis. Filamentous Gram-positive aggregates within granulation tissue and abscess, without malignancy or granulomas, were reported as suggestive of actinomycosis [1,2,4]. Culture of *Actinomyces* is often negative [1,2]. Yield falls further after β-lactam therapy; even a single dose of an active antibiotic can sterilise sputum or lavage while organisms persist in tissue. Other factors include the need for anaerobic conditions, incubation for 10-20 days and overgrowth by companion flora [2]. This patient had already received intravenous co-amoxiclav for two days and oral co-amoxiclav for eight weeks before the relevant specimens were obtained. Co-amoxiclav is active against *Actinomyces* species, so a negative culture was expected and does not exclude the diagnosis [1,2].

*Aggregatibacter actinomycetemcomitans* and *E. corrodens* were isolated only from enrichment cultures. Both are oral *Haemophilus*, *Aggregatibacter*, *Cardiobacterium*, *Eikenella* and *Kingella* (HACEK) commensals and may appear as co-pathogens in polymicrobial soft tissue infection [5-7]. *Aggregatibacter actinomycetemcomitans* can itself cause invasive thoracic and chest wall disease [6,8,9]. Recovery from enrichment culture alone does not establish pathogenicity and does not confirm actinomycosis. Their clinical significance here is uncertain.

Laboratory trends fit the clinical course. Marked neutrophilia, reactive thrombocytosis and a C-reactive protein peaking at 186 mg/L indicated severe bacterial infection. The sustained fall in C-reactive protein, normalisation of the white cell count and recovery of haemoglobin provided objective evidence that prolonged antimicrobial therapy had controlled the infection.

Thoracic actinomycosis is usually treated with a prolonged β-lactam course because of fibrosis and abscess formation [1,2]. This patient received intravenous co-amoxiclav 1.2 g for two days, then oral co-amoxiclav 625 mg three times daily for eight weeks, then oral cefalexin 1 g twice daily for four months, giving a total course of about six months.

An important limitation is that *Actinomyces* was not recovered in culture and the histology was only suggestive. The diagnosis therefore remained presumptive. It was supported by pulmonary-to-chest wall extension, histology suggestive of *Actinomyces*, investigations that made tuberculosis and malignancy substantially less likely and a sustained response to prolonged antimicrobial therapy [2,4]. These features may justify continued treatment but do not establish a culture-proven diagnosis.

Thoracic actinomycosis should be considered when chronic or recurrent pulmonary consolidation extends into the chest wall, particularly after tuberculosis has become substantially less likely [2,3]. Early tissue sampling for histology and prolonged anaerobic culture, with close microbiological liaison, remains essential. Prior antibiotics are a common reason for a negative *Actinomyces* culture and should not, on their own, lead to the diagnosis being discarded [2].

## Conclusions

Thoracic actinomycosis is an uncommon but important cause of chronic pulmonary and chest wall infection. Its ability to cross anatomical tissue planes can produce a mass-like lesion that closely mimics tuberculosis or malignancy.

In this young woman, recurrent left-sided pneumonia, haemoptysis, weight loss and a large chest wall mass initially raised strong suspicion of tuberculosis. Extensive mycobacterial investigations were negative. Histology demonstrating filamentous Gram-positive aggregates suggestive of *Actinomyces*, supported by the isolation of *Aggregatibacter actinomycetemcomitans* and *Eikenella corrodens*, supported a presumptive diagnosis of thoracic actinomycosis. Prolonged antibiotic therapy led to clinical and biochemical resolution. This case highlights the need to maintain a broad differential diagnosis and to obtain adequate tissue for both histological and microbiological examination when chronic pulmonary disease extends into the chest wall.
